# Structural Design and Performance Evaluation of a Janus Silica-Based Nanosheet Composite Viscosity Reducer

**DOI:** 10.3390/molecules31122061

**Published:** 2026-06-12

**Authors:** Jingchun Wu, Bo Li, Fang Shi, Yang Zhao, Miaoxin Zhang, Liyuan Cai, Fengshan Guo, Chunlong Zhang

**Affiliations:** 1 Key Laboratory for EOR Technology (Ministry of Education), Northeast Petroleum University, Daqing 163318, China; nepu_li@163.com (B.L.); sfang1916@163.com (F.S.); dqzy0683@163.com (Y.Z.); sygc8810@163.com (M.Z.); sygc9212@163.com (L.C.); fantast_17@163.com (F.G.); 2 Daqing Yongzhu Petroleum Technology Development Co., Ltd., Daqing 163000, China; zhangchunlong0505@outlook.com

**Keywords:** high waxy heavy oil, chemical cold production, nano viscosity reducer, synergistic effect

## Abstract

Aiming at the characteristics of high viscosity and poor fluidity of high waxy ordinary heavy oil, a Janus silica-based nanosheet composite viscosity reducer was designed and prepared in this paper. The viscosity reducer was assembled by asymmetric Gemini viscosity reducer and silica nanosheets through dehydration condensation reaction, and its structure was verified by FT-IR, ^1^HNMR, XPS and DLS. The viscosity reduction performance, emulsion stability, interfacial tension and flow performance of the viscosity reducer were systematically evaluated by taking heavy oil with wax content of 35.7% and viscosity of 237 mPa·s at 30 °C as the research object. The results showed that, at an oil-to-viscosity-reducer-solution volume ratio of 3:7 and a viscosity reducer mass fraction of 0.3%, the maximum viscosity reduction rate reached 94.5% at 30 °C, calculated relative to the viscosity of the dehydrated original heavy oil. The oil–water interfacial tension was significantly reduced, and the 24 h bleeding ratio, defined as the volume percentage of separated water relative to the initial aqueous phase volume, was only 7.3%, indicating good emulsion stability. The core flow experiment shows that the resistance coefficient is reduced to the lowest at 0.3% concentration, and the seepage capacity is significantly improved. The analysis of total hydrocarbon gas chromatography showed that the content of high-carbon wax components in the C_23_-C_30_ range decreased by 4.79 percentage points after treatment, indicating that the viscosity reducer preferentially interacted with high-carbon wax molecules and promoted wax-crystal dispersion, thereby weakening the three-dimensional wax-crystal network. The viscosity reducer has the synergistic effect of dispersing wax crystals, reducing interfacial tension and stabilizing emulsification, which provides a low-cost and high-performance technical approach for the efficient exploitation of high waxy ordinary heavy oil.

## 1. Introduction

With the continuous reduction of global light crude oil reserves, as an important alternative energy source, the efficient development of heavy oil has become a key measure to ensure global energy security [1]. According to the latest resource census data, the proportion of heavy oil in global oil resources is about 70%. Among them, the heavy oil resources in the United States reach 12 billion tons [2,3,4], the proven heavy oil reserves in China exceed 3.5 billion tons, and the proven reserves of offshore heavy oil reach 600 million tons, accounting for 20% of the proven reserves of heavy oil in China, with great development potential [5,6]. At present, heavy oil thermal recovery technology is the most widely used. In 2025, the thermal recovery production exceeded 1.3 million tons. It is expected that the annual thermal recovery production will jump to 2 million tons in 2026 [7,8,9]. The cold production technology has also made breakthrough progress [10,11]. Through direct cold investment, hot water viscosity reduction and other processes, the cost is reduced by 3.2 million yuan, and the production time rate is increased by 5% year-on-year [12,13]. Chemical viscosity reduction technology has become an important supplementary means for medium and low temperature heavy oil exploitation due to its convenient construction and controllable cost. It is often used in conjunction with thermal recovery and cold recovery technologies [14,15,16]. However, the existing technology still faces significant bottlenecks: thermal recovery technology has high energy consumption, high cost, and is easy to cause problems such as steam channeling and reservoir damage [17]; the cold production technology is only suitable for heavy oil with a specific viscosity range, and the viscosity reduction effect on high waxy heavy oil is limited, which is difficult for meeting the needs of large-scale development [18].

**Figure 1:**
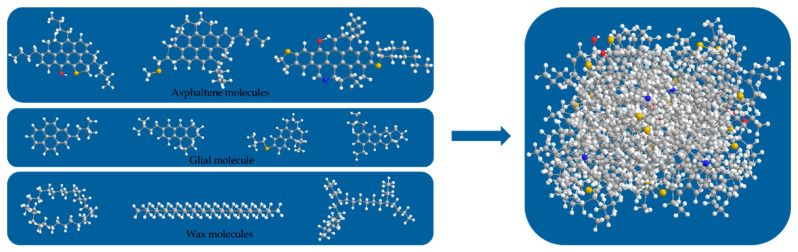
Schematic diagram of viscous mechanism of heavy oil.

This paper focuses on ordinary heavy oil with high wax content. The average wax content is 35.7%, the total content of resin and asphaltene is 21.8%, the viscosity is 237 mPa·s under formation conditions (30 °C), and the density is 0.899 g/cm^3^ underground conditions (20 °C). From the perspective of composition, wax crystals are easy to precipitate and aggregate when they are close to or below the wax precipitation point under formation or room temperature conditions [19,20]. A large number of needle/flake wax crystals are formed by entanglement of van der Waals force and molecular chains, and they overlap with each other to form a three-dimensional network gel structure, which encapsulates and binds the resin, asphaltene and oil phase in crude oil [21,22]. At the same time, the wax crystal network significantly increases the resistance of molecular motion inside the system, resulting in a sharp decline in the fluidity of crude oil, which is manifested by a significant increase in viscosity, a rise in freezing point, and a very poor low-temperature fluidity, which eventually causes heavy oil flow difficulties [23,24]. In addition, resins and asphaltenes form supramolecular aggregates and three-dimensional network structures through strong hydrogen bonds, π-π stacking and polar interactions, which further increase internal friction resistance and structural viscosity and aggravate the viscosity of heavy oil [25,26,27], as shown in Figure 1.

For high-waxy heavy oil, the major bottleneck of cold production is the formation of a three-dimensional wax-crystal network at relatively low temperature, which severely restricts oil flowability. Thermal recovery can reduce viscosity by increasing temperature and dissolving or melting wax crystals; however, it is usually accompanied by high energy consumption, steam channeling, heat loss, and potential reservoir damage. Chemical viscosity reduction provides an alternative low-energy approach, mainly through wax-crystal dispersion, interfacial tension reduction, and emulsification. Nevertheless, conventional chemical viscosity reducers often fail to simultaneously achieve efficient wax inhibition, strong interfacial activity, stable emulsion formation, and improved flow performance in porous media.

Therefore, this paper aims to synthesize a Janus silica-based nanosheet composite viscosity reducer through the assembly of silicon dioxide nanosheets and asymmetric Gemini viscosity reducer to achieve efficient viscosity reduction of high waxy ordinary heavy oil. After the completion of preparation, the synthesis effect of Janus silica-based nanosheet composite viscosity reducer was verified by FT-IR, ^1^HNMR, XPS and DLS characterization methods. In addition, this study focuses on the key viscosity-forming processes of high-waxy heavy oil, including wax-crystal network formation, oil–water interfacial resistance, and emulsion instability. The viscosity-reduction mechanism of the Janus silica-based nanosheet composite viscosity reducer was verified through viscosity reduction rate, emulsion stability, oil–water interfacial tension, core flooding performance, and changes in wax-component distribution.

## 2. Results and Discussion

### 2.1. Characterization

#### 2.1.1. Result of Fourier Transform Infrared Spectrum

**Figure 2:**
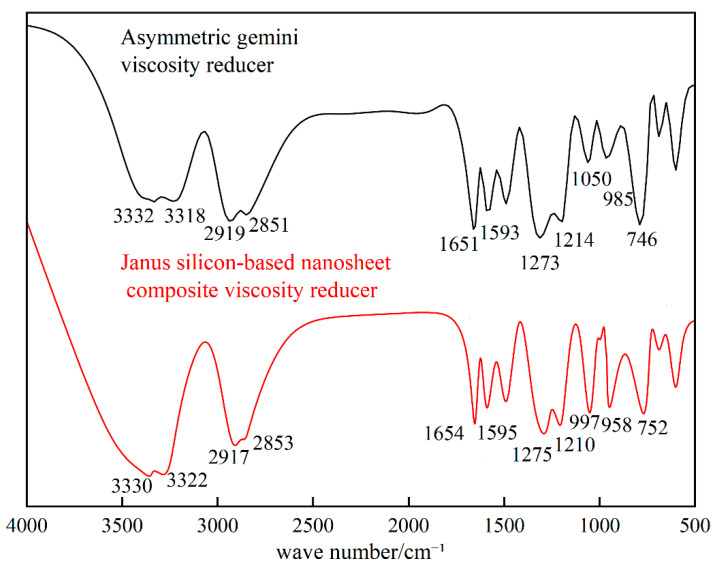
FT-IR spectra.

Figure 2 shows the FT-IR spectral characteristics of the target product. Among them, the wide and strong absorption peak at 3330 cm^−1^ is attributed to the stretching vibration of hydroxyl group (-OH), and its peak broadening reflects the existence of hydrogen bonding between hydroxyl groups, which confirms that hydroxyl groups are successfully introduced on the C_2_ spacer. The characteristic peaks at 3322 cm^−1^ and 1654 cm^−1^ correspond to the N-H stretching vibration and C=O stretching vibration of the amide group (-CONH-), respectively, indicating that the long-chain alkanes are stably connected to the benzene ring through the amide bond. The absorption peaks at 2917 cm^−1^ and 2853 cm^−1^ are attributed to the asymmetric and symmetric stretching vibrations of -CH_2_- in the long-chain alkane, confirming that the molecule contains sufficient alkyl chains. The absorption peaks at 1595 cm^−1^ and 752 cm^−1^ are derived from the stretching vibration of the benzene ring skeleton and its C-H stretching vibration, respectively, confirming the existence of the benzene ring structure. The strong absorption peaks at 1275 cm^−1^ and 1210 cm^−1^ are the stretching vibration peaks of S=O in the sulfate group (-OSO_3_-), and the stretching vibration peak of S-O at 997 cm^−1^, indicating that the sulfate group was successfully grafted. In addition, the absorption peak at 958 cm^−1^ is attributed to the Si-O stretching vibration, confirming that the silica-based nanosheets have been successfully connected to the molecular skeleton.

#### 2.1.2. Result of Nuclear Magnetic Resonance of Hydrogen

**Figure 3:**
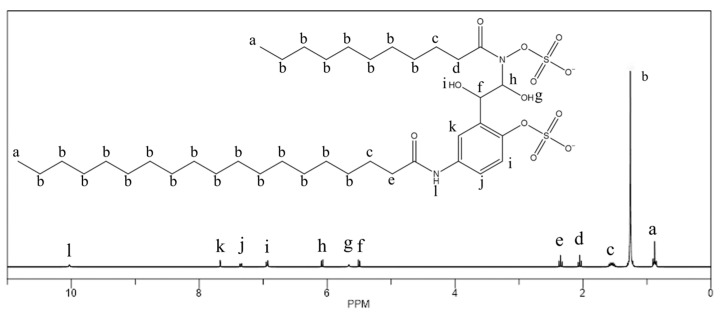
Nuclear magnetic resonance spectrum of hydrogen. The different letters represent labels for distinct hydrogen environments, corresponding to proton signal assignments in the molecule.

In the ^1^H NMR spectra of the target viscosity reducer, the chemical shift, peak shape, integral area and coupling splitting characteristics of each functional group are consistent with the molecular design structure (Figure 3). The integral ratio of the characteristic peaks of methyl and methylene in the hydrophobic chain is consistent with the number ratio of hydrogen atoms in the C_18_/C_10_ asymmetric chain. The broad peak characteristics of phenolic hydroxyl group and spacer hydroxyl group confirmed the existence of an intermolecular hydrogen bond network. The characteristic peaks of the benzene ring and the C_2_ spacer appeared completely, indicating that the connection reaction between the C_2_ spacer and the benzene ring skeleton was complete. No residual signal of raw materials was detected in the spectrum, which proved that the conversion rate of the synthesis process was high and the structure of the target product was correct.

#### 2.1.3. Result of X-Ray Photoelectron Spectrum

**Figure 4:**
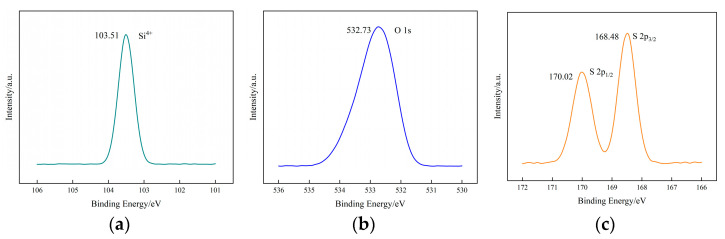
X-ray photoelectron spectroscopy. (**a**) High-resolution Si 2p spectrum; (**b**) High-resolution O 1s spectrum; (**c**) High-resolution S 2p spectrum.

The Si 2p XPS spectrum shown in Figure 4a shows a single symmetrical characteristic peak at 103.51 eV, which belongs to the Si^4+^ species in the SiO_2_ framework. Compared with pure silica, the binding energy of this peak shifts positively by about 0.3 eV, indicating that the chemical environment around silicon atoms changes due to the formation of Si-O-S covalent bonds.

The Figure 4b O 1s spectrum can be fitted into three characteristic components, which are attributed to Si-O-Si (532.73 eV), Si-O-S/S = O (532.33 eV) and Si-OH (533.57 eV), respectively. The appearance of the new Si-O-S/S = O component and the decrease in the relative content of Si-OH confirmed the dehydration condensation reaction between the silicon hydroxyl group and the sulfate group.

The spectrum of S 2p in Figure 4c shows a typical spin–orbit splitting double peak. S 2p_3/2_ and S 2p_1/2_ are located at 168.48 eV and 170.02 eV, respectively. The splitting energy is 1.5 eV, which is completely consistent with the characteristics of sulfur species in the Si-O-SO_2_-O-covalent structure. It is directly proved that the asymmetric Gemini viscosity reducer molecules are stably grafted onto the surface of silica through the Si-O-S covalent bond.

#### 2.1.4. Result of Dynamic Light Scattering

**Figure 5:**
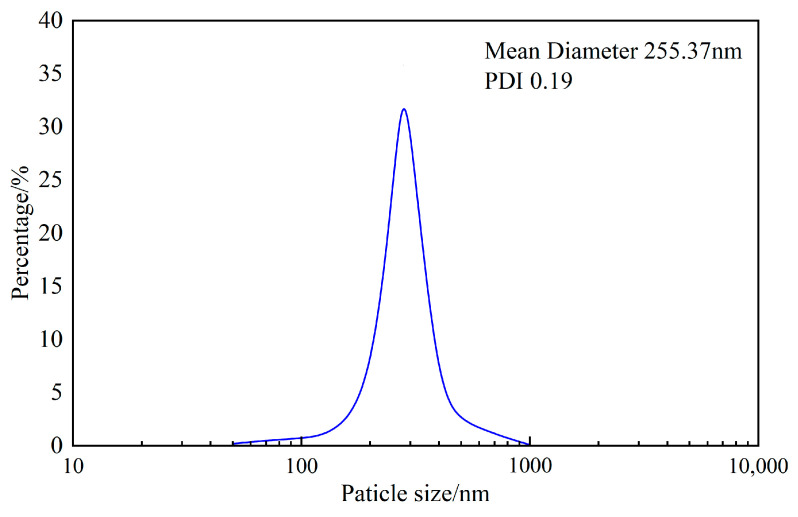
Size distribution of target viscosity reducer.

The hydrodynamic particle size and dispersion of the prepared Janus silica-based nanosheet composite viscosity reducer were characterized by dynamic light scattering (DLS). As shown in Figure 5, the sample showed a single-peak narrow distribution in the aqueous phase, the average hydrodynamic particle size was 255.37 nm, and the polydispersity index (PDI) was 0.19, indicating that the nanosheets were uniform in size and well dispersed.

### 2.2. Performance Evaluation

#### 2.2.1. Result of Viscosity Reduction Performance Evaluation

**Figure 6:**
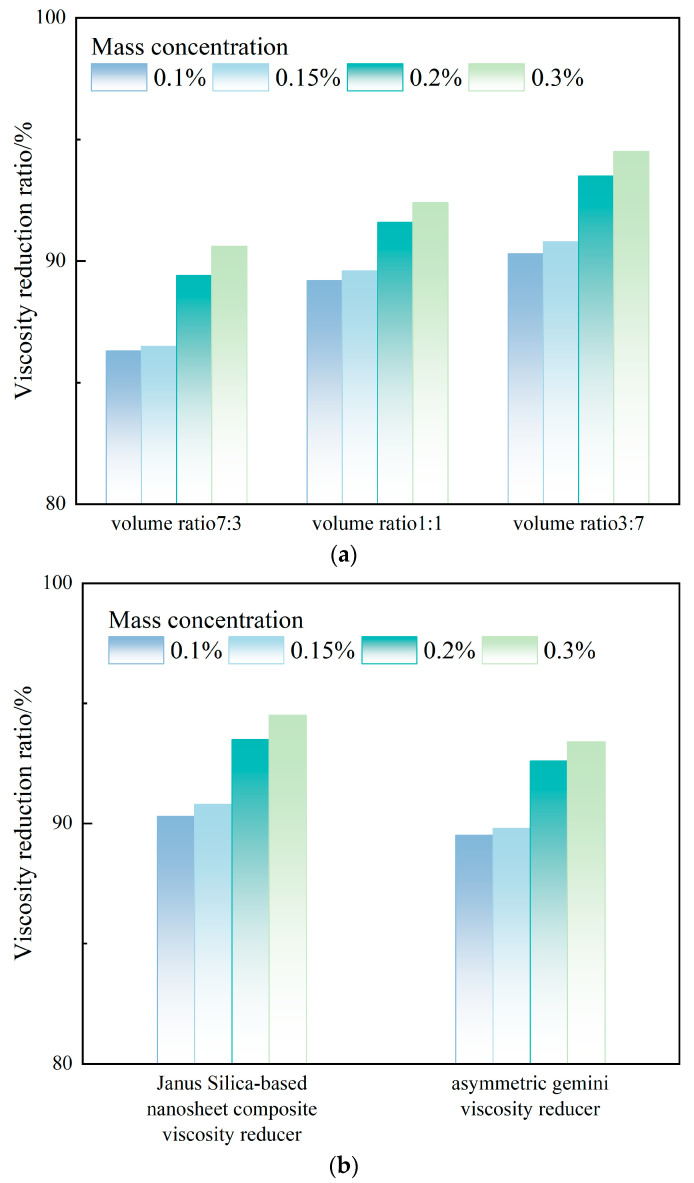
Viscosity reduction performance evaluation diagram. (**a**) Viscosity reduction rates of the viscosity reducer under different conditions; (**b**) Viscosity reduction rates of the viscosity reducer before and after grafting silica nanosheets at different concentrations.

The experimental results show that the viscosity reducer has a significant viscosity reduction effect on heavy oil, and the maximum viscosity reduction rate can reach 94.5%. The oil-to-viscosity-reducer-solution volume ratio is the key factor affecting the viscosity reduction performance. Under the same mass fraction, the viscosity reduction rate when the oil-to-viscosity-reducer-solution volume ratio is 3:7 is higher than 1:1 and 7:3. At the same time, in the case of a fixed oil-to-viscosity-reducer-solution volume ratio, as the mass fraction of the viscosity reducer increases from 0.1% to 0.3%, the viscosity reduction rate shows an upward trend, and the rising rate shows a slow–fast–slow change rule. Among them, the rising rate is the fastest in the range of 0.15% to 0.2%, indicating that the mass fraction of the viscosity reducer is also an important factor affecting the viscosity reduction effect (Figure 6a).

The viscosity reduction rate was calculated using the viscosity of the dehydrated original heavy oil at 30 °C as the baseline. All viscosity measurements were performed in triplicate under identical conditions. The result of 94.5% corresponds to the oil-to-viscosity-reducer-solution volume ratio of 3:7 and a viscosity reducer mass fraction of 0.3%.

At the oil-to-viscosity-reducer-solution volume ratio of 3:7, the Janus silica-based nanosheet composite viscosity reducer was compared with the asymmetric Gemini viscosity reducer without assembled silica nanosheets at different concentration gradients. The viscosity reduction rate results are shown in Figure 6b. The results show that the viscosity reduction rate of the viscosity reducer assembled with silica nanosheets is higher than that of the asymmetric Gemini viscosity reducer, which indicates that the silica nanosheets make the viscosity reducer molecules more orderly arranged at the oil–water interface and improve the viscosity reduction effect.

#### 2.2.2. Result of Emulsifying Performance Evaluation

**Figure 7:**
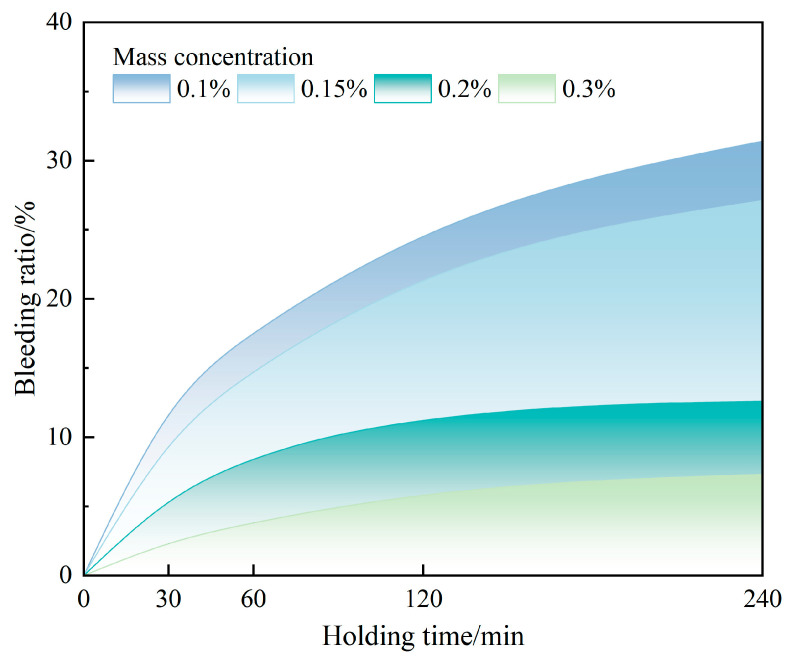
Bleeding ratio diagram under different concentrations.

Under the condition of oil-to-viscosity-reducer-solution volume ratio of 3:7, the emulsification performance evaluation results show that the viscosity reducer can effectively improve the oil–water emulsification state, and its emulsification stability is closely related to the mass fraction of the viscosity reducer (Figure 7). With the extension of standing time, the bleeding ratio of emulsions under the action of different concentrations of viscosity reducers gradually increased. Among them, the emulsion stability was poor at 0.1% and 0.15% concentrations, and the bleeding ratios after standing for 24 h were about 31.4% and 27.1%, respectively. The stability of the emulsion at 0.2% and 0.3% concentrations was significantly improved, and the 24 h bleeding ratio decreased to about 12.6% and 7.3%, respectively, which was significantly lower than that of the low concentration group.

**Figure 8:**
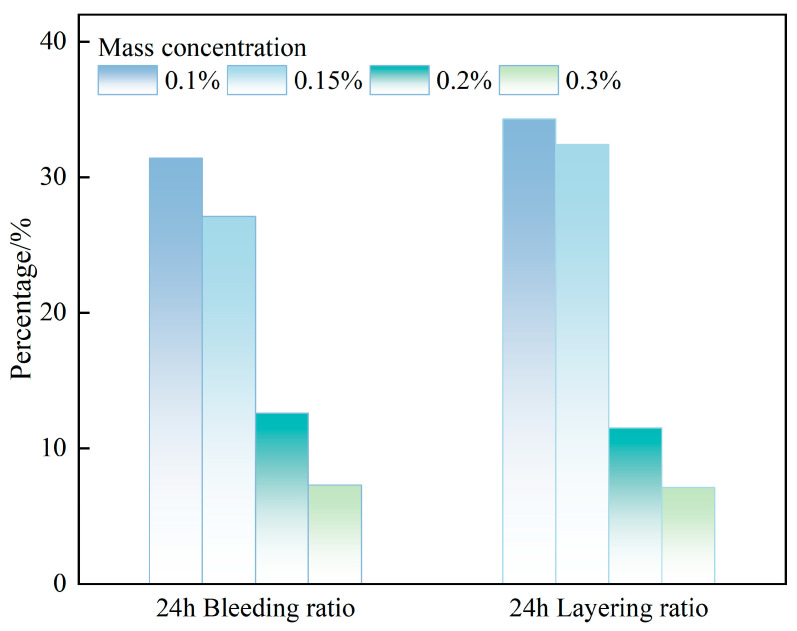
Comparison of 24 h bleeding ratio and layering ratio.

It can also be seen from the stratification ratio results of 24 h that the layering ratio of 0.2% and 0.3% concentration groups is much lower than that of 0.1% and 0.15% concentration groups, indicating that under the experimental conditions, 0.2% and 0.3% viscosity reducers are more likely to form stable emulsions with the target heavy oil (Figure 8).

#### 2.2.3. Result of Interfacial Tension Testing

**Figure 9:**
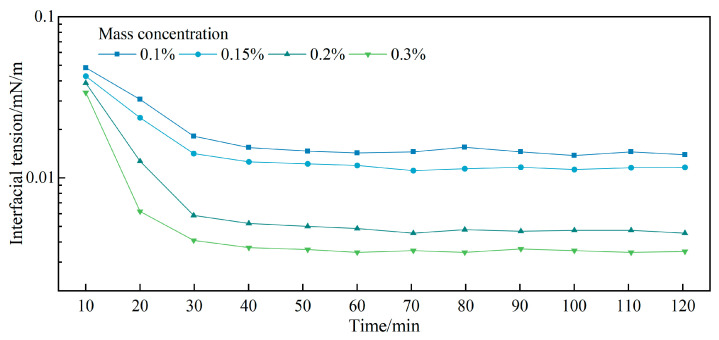
Interface tension diagram at different concentrations.

Figure 9 shows that the prepared Janus silica-based nanosheet composite viscosity reducer can significantly reduce the oil–water interfacial tension, and the interfacial tension decreases significantly with the increase of the mass fraction of the viscosity reducer. The interfacial tension is greatly reduced when the mass fraction of viscosity reducer is 0.1% and 0.15%. When the mass fraction increases to 0.2% and 0.3%, the interfacial tension further decreases and tends to be stable, both at a low level, which is conducive to the emulsification and dispersion of crude oil. In general, 0.3% and 0.5% are the preferred mass fractions of the viscosity reducer, which can achieve excellent interfacial activity and reduce interfacial tension.

#### 2.2.4. Result of Fluidity Evaluation

**Figure 10:**
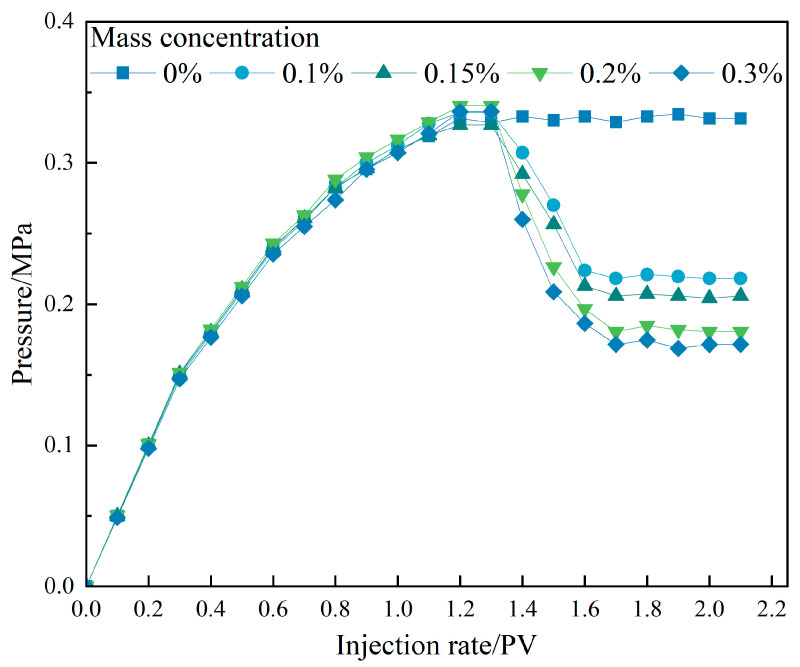
Pressure variation curve with injection rate.

The results of core flow experiments show that when the viscosity reducer concentration is 0%, the resistance coefficient is 1, the flow resistance of the system does not change, and the core seepage characteristics remain basically stable. With the increase in the concentration of the viscosity reducer, the flow resistance of the system is significantly reduced, and the resistance coefficient at each concentration is less than 1, indicating that the viscosity reducer can effectively reduce the viscosity of heavy oil, reduce the seepage resistance of the oil viscosity reducer mixture in the core, thereby improving the fluid flow capacity. When the concentration of viscosity reducer is 0.3%, the resistance coefficient is the lowest, and the effect of viscosity reduction and drag reduction is the best (Table 1, Figure 10).

#### 2.2.5. Chromatographic Analysis Result of Petroleum Total Hydrocarbon

**Figure 11:**
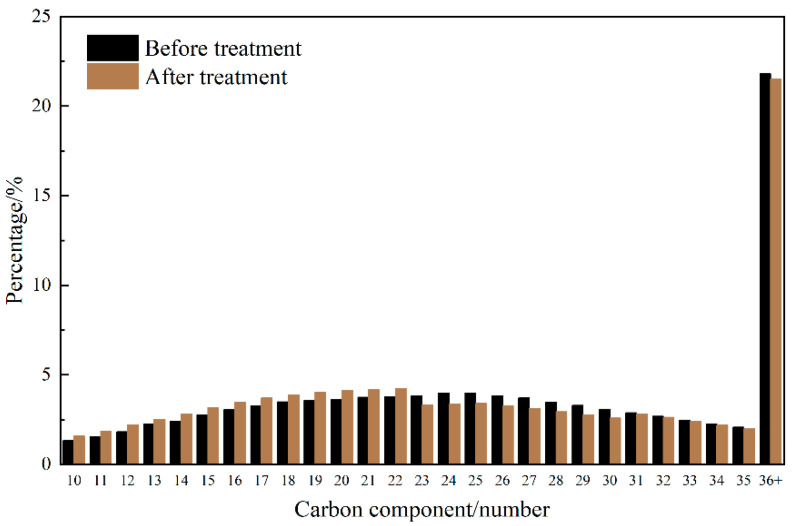
Total hydrocarbon analysis diagram.

The total hydrocarbon carbon number distribution analysis results are shown in Table 2 and Figure 11. In the original heavy oil, the total content of C_10_-C_35_ carbon number hydrocarbons is 78.20%, and the C_36+_ heavy component is 21.80%.

After viscosity reducer treatment, the total content of C_10_-C_35_ was 78.50%, and C_36+_ was 21.50%. With the original heavy oil. Compared with the crude oil, the content of wax components (C_23_-C_35_) in the treated crude oil decreased by 4.79 percentage points, among which the reduction of high carbon wax (C_23_-C_30_) was the most significant.

The change in carbon-number distribution after treatment should not be interpreted as chemical cracking or degradation of hydrocarbons. Under the mild experimental conditions used in this study, the viscosity reducer mainly changes the physical distribution and aggregation state of wax-rich components. The long hydrophobic chains of the asymmetric Gemini molecule can interact with high-carbon wax molecules, especially those in the C_23_-C_30_ range, through hydrophobic association and van der Waals interactions. This interaction promotes the adsorption of viscosity-reducer molecules onto wax-crystal surfaces, disturbs the ordered packing and growth of wax crystals, and reduces wax-crystal aggregation. Therefore, large interconnected wax-crystal aggregates are converted into smaller and more isolated wax-rich domains. The decrease in the relative content of C_23_-C_35_ components in the separated oil phase provides indirect evidence for the preferential physical interaction between the viscosity reducer and high-carbon wax components.

### 2.3. Proposed Mechanism of Synergistic Viscosity Reduction

Based on the above results, the viscosity reduction of high-waxy heavy oil by the Janus silica-based nanosheet composite viscosity reducer can be attributed to the synergistic effects of wax-crystal dispersion, interfacial tension reduction, and emulsion stabilization. First, the long alkyl chains in the asymmetric Gemini viscosity reducer show strong affinity for high-carbon wax molecules. Specifically, the dispersion of wax crystals is achieved through adsorption-induced surface modification. The long alkyl chains of the asymmetric Gemini viscosity reducer can adsorb onto wax-crystal surfaces through hydrophobic association and van der Waals interactions with high-carbon wax molecules. After adsorption, the asymmetric molecular structure interferes with the regular packing and continuous growth of wax crystals. Meanwhile, the exposed amide, hydroxyl and sulfate groups introduce steric hindrance, electrostatic repulsion and wettability regulation, which reduce direct contact and bridging between wax crystals. The Janus silica-based nanosheets can further act as interfacial physical barriers, suppressing the collision and reaggregation of wax crystals. As a result, large interconnected wax-crystal aggregates are transformed into smaller and more isolated wax-rich domains, leading to a weakened three-dimensional wax-crystal network. Their adsorption or co-assembly on wax-crystal surfaces can interfere with the ordered arrangement of wax molecules and inhibit the formation of a continuous three-dimensional wax-crystal network. Second, the amphiphilic Gemini structure facilitates adsorption at the oil–water interface and significantly reduces the oil–water interfacial tension, thereby promoting the dispersion of heavy oil into smaller droplets. Third, the Janus silica-based nanosheets can be anchored at the oil–water interface. The grafted organic chains enhance oil-phase affinity, whereas the retained Si–OH groups improve water-phase dispersibility. This asymmetric wettability allows the nanosheets to form a stable interfacial protective layer, improving Pickering emulsion stability and reducing water separation. As a result, the internal structural resistance of the heavy oil system and the seepage resistance in porous media are both reduced, which is consistent with the viscosity reduction, interfacial tension, emulsion stability, and core flooding results.

## 3. Materials and Methods

### 3.1. Materials

Tetraethyl orthosilicate (98%), hydrophobic silica nanoparticles modified by hexamethyldisilazane, native average particle size of 20 nm, cyclohexane (≥99.5%), anhydrous pyridine (99.8%), paraffin (melting point 58–60 °C) were purchased from Sigma-Aldrich (St. Louis, MO, USA); chlorosulfonic acid (99%) for the activation of sulfate groups in viscosity reducer molecules; ammonia (25–28 wt % aqueous solution), dichloromethane (≥99.9%), absolute ethanol (≥99.7%), and carbon tetrachloride (≥99.5%) were purchased from National Pharmaceutical Group Chemical Reagents Co., Ltd. (Shanghai, China); deionized water (laboratory-made) and polyvinylidene fluoride needle filter, aperture 0.22 μm, diameter 25 mm, were purchased from Jinteng Experimental Equipment Co., Ltd. (Tianjin, China). The asymmetric Gemini viscosity reducer used in this study was self-made in the laboratory. The molecular structure of the viscosity reducer was confirmed by nuclear magnetic resonance hydrogen spectrum. The other reagents were analytically pure and directly used without purification. The heavy oil used in this study is high waxy ordinary heavy oil. The wax content is 35.7%, the resin content is 21.8%, the viscosity is 237 mPa·s at 30 °C under formation conditions, and the density is 0.899 g/cm^3^ at 20 °C underground conditions.

### 3.2. Preparation of Janus Silica-Based Nanosheet Composite Viscosity Reducer and Characterization

A stable water-in-oil Pickering emulsion was obtained by high-speed shear emulsification of 70 mL cyclohexane and 30 mL deionized water using hydrophobic fumed silica nanoparticles with a mass fraction of 1.0% as stabilizer. The mixture was emulsified at 6000 rpm for 3 min using a rotor–stator dispersing head with a diameter of 18 mm (High-shear homogenizer, Model JRJ300-S, Jiangyin Poly Research Instrument Co., Ltd., Jiangyin, Jiangsu, China). During emulsification, the temperature was maintained at 30 °C. After dissolving 1.0 mL TEOS in the oil phase, dilute ammonia was added dropwise to adjust the pH of the system to 9.0–10.0, and stirred at room temperature for 12 h, so that TEOS underwent hydrolysis-condensation reaction at the oil–water interface to form a silica shell in situ. After the reaction was completed, the hollow silica microspheres were centrifuged at 8000 rpm for 10 min, washed three times with absolute ethanol and deionized water alternately, and vacuum dried at 60 °C for 12 h to obtain hollow silica microspheres with rich silicon hydroxyl groups on both inner and outer surfaces.

**Figure 12:**
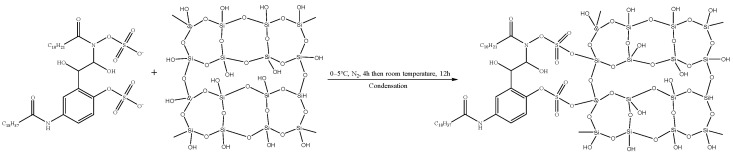
Reaction equation.

The dried hollow silica microspheres were dispersed in molten paraffin at 60 °C. After ultrasonic dispersion, the hollow silica microspheres were quickly cooled to room temperature, so that the inner cavity of the paraffin-embedded microspheres was solidified, and only the silicon hydroxyl active sites on the outer surface of the microspheres were exposed. The semi-embedded microspheres of paraffin were dispersed in anhydrous dichloromethane, and self-made viscosity reducer was added, and anhydrous pyridine was added at the same time. Under the atmosphere of 0–5 °C and nitrogen protection, chlorosulfonic acid was slowly added dropwise to activate the sulfate group. After stirring for 4 h, it slowly rose to room temperature and continued to react for 12 h. The sulfate group at the end of the viscosity reducer molecule was dehydrated and condensed with the silicon hydroxyl group on the outer surface of the microsphere to form a stable Si-O-S covalent bond (Figure 12). During the reaction, the molar ratio of silicon hydroxyl group:self-made viscosity reducer:anhydrous pyridine:chlorosulfonic acid = 1:0.3:0.36:0.33. After the reaction, the product was washed with anhydrous dichloromethane three times to remove the residual reagent, then carbon tetrachloride was added to dissolve the paraffin embedding layer, and the microspheres were collected by centrifugation. The microspheres were fully washed with anhydrous ethanol and deionized water in turn, and dried in vacuum at 40 °C. The modified hollow silica microspheres with the self-made viscosity reducer molecule covalently grafted on the outer surface and the original silicon hydroxyl group retained on the inner surface were obtained.

**Figure 13:**
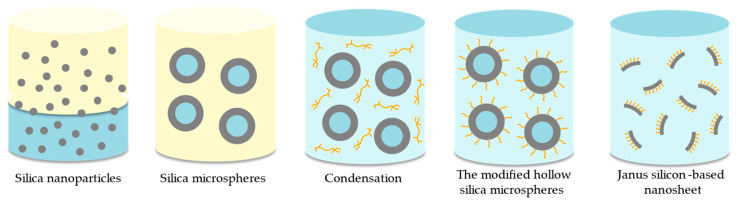
Schematic diagram of synthesis process. The gray part represents silica, and the orange part represents the asymmetric gemini viscosity reducer.

The modified hollow silica microspheres were dispersed in deionized water at a concentration of 1 mg/mL, placed in an ice water bath, and broken by a probe-type ultrasound instrument. The ultrasonic power was 200 W, 30 min, working 2 s, intermittent 3 s; after the crushing was completed, the large particles that were not completely broken were removed by centrifugation at 4000 rpm for 5 min, and the collected supernatant was the target product—Janus silica-based nanosheet composite viscosity reducer. The complete synthesis process is shown in Figure 13.

#### 3.2.1. Fourier Transform Infrared Spectrum

The WQF-410 Fourier Transform Infrared Spectrometer (Beijing Beifen-Ruili Analytical Instrument (Group) Co., Ltd., Beijing, China) was used for detection. The scanning wave number was between 4000~500 cm^−1^, and the presence of the target functional group was confirmed by the characteristic absorption peak.

#### 3.2.2. Nuclear Magnetic Resonance of Hydrogen

The samples were detected by SPEC-PMR nuclear magnetic resonance analyzer of Beijing Spike Technology Development Co., Ltd., Beijing, China, and the samples were dissolved with CDCl_3_ as a reagent. The samples were prepared into a solution with a mass concentration of 0.5 mg/mL and tested at room temperature. The chemical shift and peak area of the hydrogen nucleus were recorded, and the hydrogen atom environment and connection mode in the molecular structure were analyzed.

#### 3.2.3. X-Ray Photoelectron Spectrum

The X-ray photoelectron spectroscopy test was carried out by using the ESCALAB 250xi (11 OOV) spectrometer of Semmerfeld Technology in Waltham, MA, USA. The monochromatic Al Kα X-ray source was used, and the working power was 150 W; the full-spectrum and high-resolution spectra were collected in ultra-high vacuum environment, and the qualitative and quantitative analysis of elements and chemical state analysis were realized by analyzing the kinetic energy and intensity of photoelectrons.

#### 3.2.4. Dynamic Light Scattering

The particle size distribution was measured by Mastersizer3000 laser particle size analyzer (Malvern, UK). The temperature was 25 °C and the scattering angle was 173°.

### 3.3. Viscosity Reduction Performance Evaluation

The heavy oil sample was placed in a thermostat at 60 °C for 24 h to separate free water and then centrifuged at 3000 r/min for 30 min for further dehydration, and the initial mass of the sample was recorded. The simulated formation water with salinity of 12,000 mg/L was used to prepare viscosity reducer solutions with mass fractions of 0.1%, 0.15%, 0.2% and 0.3%, respectively. In a 250 mL beaker, the pretreated heavy oil and different concentrations of viscosity reducer solutions were added according to oil-to-viscosity-reducer-solution volume ratio of 7:3,5:5 and 3:7, respectively, and magnetically stirred at 400 r/min for 30 min to fully mix. The apparent viscosities of the initial heavy oil and the mixtures of heavy oil with the viscosity reducer solution were measured using a DV2T rotational viscometer (Brookfield Engineering Laboratories, Inc., Middleboro, MA, USA). Before measurement, the samples were allowed to stand to remove entrained air bubbles and pre-equilibrated at 30 °C for 20 min. To ensure consistent shearing conditions, all viscosity measurements were conducted at 30 °C using spindle #0 and #1 at a constant rotational speed of 6 rpm. (Formula (1)). Each sample was measured three times, and the average value was used for analysis. The relative deviation of repeated measurements was less than 3%. The optimal conditions were determined by comparing the viscosity reduction rates at different concentrations, and the performance of the synthesized Janus silica-based nanosheet composite viscosity reducer was compared with the laboratory-made asymmetric Gemini viscosity reducer.

(1) $$\eta =\frac{\mu_{0}-\mu }{\mu_{0}}\times 100\%$$

In the formula, η is the viscosity reduction rate, %; $\mu_{0}$ is the initial viscosity of heavy oil, mPa·s; μ for the viscosity of the treated system, mPa·s.

### 3.4. Emulsifying Performance Evaluation

The type of emulsion was observed by bottle test, and the water separation rate at different times was recorded to evaluate the stability of the emulsion. The pretreated heavy oil was mixed with the viscosity reducer solution at a ratio of 3:7, and the mass fractions of the viscosity reducer solution were 0.1%, 0.15%, 0.2% and 0.3%, respectively. The mixed system was placed in a 100 mL beaker, fully emulsified by magnetic stirring at 400 r/min for 20 min, and then placed in a thermostat at 30 °C. The volume of precipitated water was recorded at 0, 30, 60, 120 and 240 min, respectively, and the emulsion stability was evaluated according to the stratification after standing for 24 h. Each group of experiments was repeated 3 times, and the average value was taken to determine the optimal concentration.

### 3.5. Interfacial Tension Testing

The pretreated heavy oil was used as the oil phase, and the viscosity reducer solutions with mass fractions of 0.1%, 0.15%, 0.2% and 0.3% were used as the water phase. The heavy oil was injected into a high-pressure quartz sample tube pre-filled with a viscosity reducer solution by a micro-syringe to form oil droplets with a diameter of 2~3 mm. The sample tube was installed on the CNG-700 interfacial tension meter (Beijing Shengwei Technology Co., Ltd., Beijing, China), and the constant temperature system was set to 30 °C. The high-speed rotation was started at 5000 r/min, so that the oil droplets were stretched into a stable columnar shape, and the droplet image was collected in real time through the high-definition camera. After the temperature was balanced, the images of each group were recorded every 10 min to monitor the morphological changes in the droplets in real time. The aspect ratio of the droplet is analyzed by software, and the interfacial tension is calculated by the built-in algorithm of the instrument, and then the change rule of interfacial tension under different conditions is investigated.

### 3.6. Fluidity Evaluation

**Figure 14:**
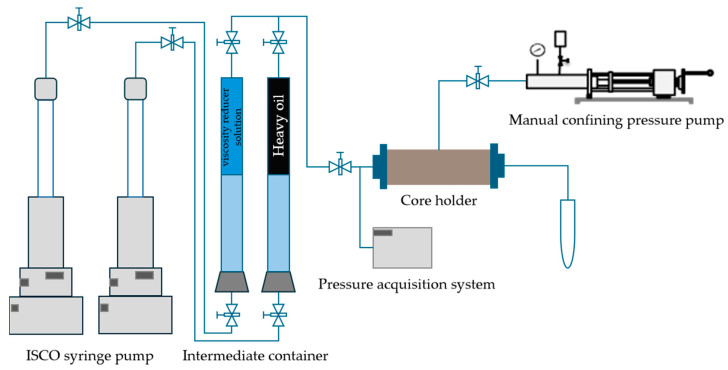
Schematic of fluidity evaluation apparatus.

Core flooding experiments were conducted using artificial cores under constant-temperature conditions. A core with dimensions of Φ2.5 cm × 10 cm was used, with a porosity of 30–35% and a gas permeability of approximately 1000 × 10^−3^ μm^2^. Before each experiment, the core was vacuum-saturated with simulated formation water for 2 h, and the initial water permeability was measured. To achieve core saturation, heavy oil and simulated formation water were co-injected at a constant flow rate with an oil-to-water flow rate ratio of 3:7. That is, the flow rate of oil was 0.3 mL/min and the flow rate of water was 0.7 mL/min. After saturation, the core was placed in a 30 °C incubator for 24 h. In the fluidity evaluation test, heavy oil and viscosity reducer solution were co-injected at a constant flow rate with an oil-to-viscosity-reducer-solution flow rate ratio of 3:7. That is, the flow rate of oil was 0.3 mL/min, and the flow rate of viscosity reducer solution was 0.7 mL/min. The schematic diagram of the experimental process is presented in Figure 14.

The mass fractions of the viscosity reducer solution tested were 0%, 0.1%, 0.15%, 0.2%, and 0.3%, respectively. The pressure drop across the core was monitored continuously. A steady state was considered to be reached when the pressure fluctuation was less than 5% within 30 min. The maximum stable pressures recorded during the oil–water co-injection and oil-viscosity-reducer-solution co-injection processes were defined as P_1_ and P_2_, respectively. Each core flooding test was repeated at least twice under the same experimental conditions, and the average value was used for analysis. Based on the collected experimental data, dynamic pressure curves were plotted, and the resistance coefficient was calculated to evaluate the flow performance of the viscosity reducer under formation-like seepage conditions (Formula (2)).

(2) $$RF=\frac{P_{2}}{P_{1}}$$

In the formula, *RF* is the resistance coefficient, %; *P*_1_ is the stable pressure during the oil–water co-injection process, MPa; and *P*_2_ is the stable pressure during the oil-viscosity-reducer-solution co-injection process, MPa.

### 3.7. Chromatographic Analysis of Petroleum Total Hydrocarbon

Total hydrocarbon gas chromatography analysis was performed according to the oil and gas industry standard SY/T 5779-2024 [28] ‘Petroleum and sedimentary organic matter hydrocarbon gas chromatography analysis method’. The blank heavy oil samples without viscosity reducer and the treated oil samples with 0.2% viscosity reducer after full stirring and centrifugal separation were determined respectively. The chromatographic data were collected by full hydrocarbon scanning mode, and the relative content of each component was calculated by peak area normalization method. By comparing the carbon number distribution of heavy oil samples before and after viscosity reducer treatment, the variation characteristics of its components were analyzed.

## 4. Conclusions

In this paper, a high-efficiency viscosity reducer was designed for high-wax ordinary heavy oil. By covalently assembling the asymmetric Gemini viscosity reducer on the surface of silica nanosheets, a Janus silica-based nanosheet composite viscosity reducer was successfully synthesized.

The results showed that the viscosity reduction rate of the composite viscosity reducer was up to 94.5% when the oil-to-viscosity-reducer-solution volume ratio was 3:7 and the mass fraction of the viscosity reducer was 0.3%, which was significantly better than the traditional viscosity reducer. The long alkyl chains promoted adsorption onto high-carbon wax molecules, while the polar groups and Janus silica-based nanosheets provided steric hindrance, electrostatic repulsion and interfacial physical barriers, thereby promoting wax-crystal dispersion and weakening the three-dimensional wax-crystal network. The Si-OH group at the hydrophilic end ensures good dispersion in the aqueous phase, and the two-dimensional sheet structure further promotes the orderly assembly of the interface. The synergistic effect of the above functions achieves the synergistic viscosity reduction effect of ‘wax crystal dispersion, interfacial tension reduction and emulsion stability’.

This study provides technical support for improving oil recovery in onshore oilfields with similar complex crude oil characteristics. In addition, the design strategy based on Janus nanomaterials and asymmetric Gemini viscosity reducers can also be extended to the development of other advanced functional materials in the fields of interface science and petroleum engineering.

## Figures and Tables

**Table 1:** Experimental data of liquidity evaluation.

Oil: Viscosity Reducer	Viscosity Reducer Concentration (%)	Core Water Permeability Measurement (×10^−3^ μm^2^)	Stable Pressure of Oil–Water Co-Injection (MPa)	Stable Pressure of Oil-Viscosity Reducer Co-Injection (MPa)	Resistance Coefficient
3:7	0	735.5	0.3315	0.3315	1
	0.1	726.3	0.3356	0.2181	0.65
	0.15	746.1	0.3267	0.2058	0.63
	0.2	715.7	0.3405	0.1805	0.53
	0.3	724.6	0.3364	0.1716	0.51

**Table 2:** Total hydrocarbon analysis data.

Carbon Number	Origin (%)	After Treatment (%)	Carbon Number	Origin (%)	After Treatment (%)	Carbon Number	Origin (%)	After Treatment (%)
10	1.32	1.58	19	3.58	4.02	28	3.48	2.94
11	1.57	1.87	20	3.68	4.12	29	3.28	2.76
12	1.87	2.19	21	3.73	4.18	30	3.08	2.58
13	2.17	2.51	22	3.78	4.23	31	2.88	2.83
14	2.47	2.83	23	3.83	3.31	32	2.68	2.62
15	2.77	3.16	24	3.93	3.36	33	2.48	2.4
16	3.07	3.48	25	3.98	3.4	34	2.28	2.19
17	3.27	3.72	26	3.83	3.26	35	2.08	1.98
18	3.43	3.86	27	3.68	3.12	36+	21.8	21.5

## Data Availability

The datasets presented in this article are not readily available because this study is in the middle of the research and development phase, and the data may be adjusted later for further improvement. The current data are for reference only. Requests to access the datasets should be directed to corresponding author.
